# Extreme Heterophilic Antibody Interference in BNP Immunoassay: A Case Report and Systematic Investigation Protocol

**DOI:** 10.1002/jcla.70280

**Published:** 2026-06-10

**Authors:** Huicong Yang, Yuanhai Zheng, Zhenhua Zeng, Haimin Gao, Jinzhi Wu, Ayang Wu, Jie Lin

**Affiliations:** ^1^ Department of Clinical Laboratory Zhangzhou Affiliated Hospital of Fujian Medical University Zhangzhou Fujian Province People's Republic of China; ^2^ Department of Endocrinology and Metabolism Zhangzhou Affiliated Hospital of Fujian Medical University Zhangzhou Fujian Province People's Republic of China; ^3^ Department of Cardiothoracic Surgery Zhangzhou Affiliated Hospital of Fujian Medical University Zhangzhou Fujian Province People's Republic of China

**Keywords:** BNP, case report, false positive, HAMA, heterophilic antibody, immunoassay interference

## Abstract

**Background:**

Heterophilic antibody (HA) interference remains a persistent challenge in immunoassay diagnostics. Human anti‐mouse antibodies (HAMA) can cause spuriously elevated biomarker results, potentially leading to misdiagnosis and unnecessary clinical interventions. Brain natriuretic peptide (BNP) is critical for heart failure diagnosis, but false‐positive results due to HAMA may trigger inappropriate clinical management.

**Case Presentation:**

A 53 year‐old male with type 2 diabetes presented with markedly elevated BNP (4394–4419 pg/mL; reference < 100 pg/mL) by chemiluminescence immunoassay, despite no heart failure symptoms, normal ejection fraction (62%), and normal NT‐proBNP (55.8 pg/mL). This discordance prompted investigation for analytical interference.

**Methods:**

Serial dilution (1:4, 1:8); specific blocking with mouse IgG, goat IgG, polymeric antibodies, and RF blocker; dose–response titration of mouse IgG (200–600 μg/mL); and alternative platform comparison.

**Results:**

Serial dilution showed linear but incomplete recovery (*R* ^2^ = 0.998), excluding hook effects. Mouse IgG induced a modest but specific 15.7% reduction in apparent BNP. Even 600 μg/mL mouse IgG achieved only partial blocking (residual BNP 2240 pg/mL), indicating high‐titer HAMA exceeding standard blocking capacity.

**Conclusions:**

This case demonstrates extreme HAMA interference causing false‐positive BNP > 4000 pg/mL. Standard blocking reagents may be insufficient for high‐titer samples. When BNP is discordant with clinical status—especially with normal NT‐proBNP—laboratories must use systematic verification to prevent misdiagnosis.

## Graphical abstract

A 53 year‐old male with type 2 diabetes presented extremely elevated BNP (> 4000 pg/mL) but normal NT‐proBNP and cardiac function. Systematic verification confirmed severe heterophilic antibody (HAMA) interference in the BNP chemiluminescence immunoassay. Serial dilution exhibited linear but incomplete recovery, and only murine IgG blocking induced a specific reduction. Standard blocking reagents were insufficient even at 600 μg/mL. Discordance between BNP and NT‐proBNP is a key indicator of analytical interference, which is critical to prevent misdiagnosis and unnecessary clinical interventions.

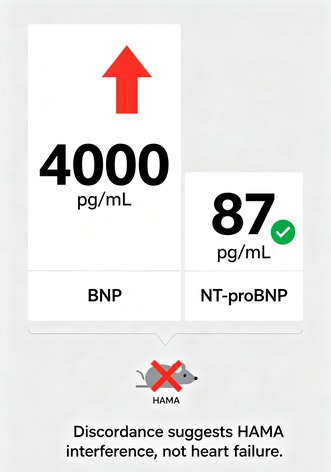

## 1 Introduction

Immunoassays using the double‐antibody sandwich format represent the analytical foundation for protein biomarker quantification [1, 2]. Despite high sensitivity and specificity, these assays are vulnerable to endogenous antibodies, particularly heterophilic antibodies and human anti‐animal antibodies (HAAA) [3, 4].

Human anti‐mouse antibodies (HAMA) are the most clinically relevant HAAA due to widespread use of murine antibodies in commercial kits [5, 6]. HAMA can bridge capture and detection antibodies, causing false‐positive signals or false‐negative results [4, 7].

Brain natriuretic peptide (BNP) and NT‐proBNP are gold‐standard biomarkers for heart failure [6, 8]. Both are measured by sandwich immunoassays and are susceptible to HAMA interference [9]. False‐positive BNP may lead to unnecessary clinical interventions, prolonged hospitalization, and patient distress [10, 11].

We report extreme HAMA interference causing spurious BNP > 4000 pg/mL in a patient with normal cardiac function and normal NT‐proBNP. This case highlights the need for vigilance for analytical interference, presents a systematic investigation approach, and demonstrates the limitations of standard blocking reagents for high‐titer HAMA.

This case also underscores the importance of appropriate test selection within the correct clinical context. Routine BNP screening in asymptomatic patients without clinical signs of heart failure may lead to spurious results and unnecessary downstream investigations.

## 2 Case Presentation

### 2.1 Clinical History and Initial Presentation

A 53 year‐old male was admitted to the Department of Endocrinology and Metabolism at Zhangzhou Affiliated Hospital of Fujian Medical University (Fujian, China) on February 24, 2026, with chief complaints of poorly controlled type 2 diabetes mellitus (diagnosed 5 years prior) and a non‐healing right foot wound of 4 months' duration. The patient reported adherence to irregular oral hypoglycemic therapy (repaglinide and acarbose) without systematic glucose monitoring.

On admission, physical examination revealed: temperature 36.5°C, pulse 85 beats/min, respiratory rate 20 breaths/min, blood pressure 107/79 mmHg. The patient appeared well‐nourished and in no acute distress. Cardiovascular examination demonstrated regular rhythm without murmurs, gallops, or rubs. Pulmonary auscultation revealed clear bilateral breath fields without rales or rhonchi. Extremity examination identified three superficial ulcerations (approximately 1 × 2 cm) on the lateral right heel with minimal crusting but no surrounding erythema, warmth, or purulent discharge. Neurological examination was unremarkable, with negative findings for diabetic peripheral neuropathy.

Initial laboratory evaluation revealed glycated hemoglobin 7.78%, indicating suboptimal glycemic control over the preceding 2–3 months. Wound culture subsequently identified *Staphylococcus aureus* , establishing the diagnosis of right diabetic foot infection (Wagner grade 3).

### 2.2 Discordant Biomarker Results

Routine cardiac assessment showed BNP 4394 pg/mL (reference < 100 pg/mL). Repeat measurements remained severely elevated. The patient had no dyspnea, edema, or heart failure signs. Cardiac troponin, ECG, and echocardiography were normal.

Most importantly, NT‐proBNP was 87.0 pg/mL (reference < 125 pg/mL), normal. The combination of extremely high BNP, normal NT‐proBNP, intact cardiac function, and no heart failure symptoms strongly suggested analytical interference rather than true BNP elevation [9, 11].

Timeline of Key Tests.

2026/2/24: Initial BNP = 4394 pg/mL.

2026/2/26: Repeat BNP = 3464 pg/mL.

2026/2/27: Echocardiography; BNP = 4011 pg/mL.

2026/2/28: NT‐proBNP = 87.0 pg/mL (normal).

2026/3/4: Systematic interference investigation.

### 2.3 Comprehensive Cardiac Evaluation

Given the profound BNP elevation without clinical correlates, comprehensive cardiac evaluation was undertaken:

Echocardiography (February 27): Normal left ventricular size and wall thickness, ejection fraction 62%, normal diastolic function, no valvular abnormalities, estimated pulmonary artery pressure within normal limits.

Chest multi‐slice CT: No cardiomegaly, pleural effusion, or pulmonary edema.

Vascular ultrasound: No significant stenosis in carotid or lower extremity arteries.

Right foot MRI: Changes consistent with diabetic foot osteomyelitis, no evidence of necrotizing fasciitis.

Most critically, NT‐proBNP measured by electrochemiluminescence immunoassay (Roche Diagnostics) on February 28 was 87.0 pg/mL (reference: < 125 pg/mL), within normal limits and completely inconsistent with the BNP results.

The combination of markedly elevated BNP, normal NT‐proBNP, intact cardiac function, and absence of heart failure symptoms strongly suggested analytical interference rather than true pathological BNP elevation [9, 11].

## 3 Materials and Methods

### 3.1 Sample Collection and Processing

Peripheral venous blood (3 mL) was collected into EDTA‐K_2_ anticoagulant tubes. Plasma was separated by centrifugation at 3000 × g for 10 min within 30 min of collection. Visual inspection confirmed absence of hemolysis, lipemia, or icterus. Samples were stored at 2°C–8°C and analyzed within 2 h to prevent BNP degradation by metalloproteases [8].

### 3.2 Immunoassay Platforms

BNP was quantified using the Pylon Immunoassay System employing a sandwich chemiluminescence immunoassay with murine monoclonal capture and detection antibodies (ET Healthcare). NT‐proBNP was measured using the Roche Cobas e601 electrochemiluminescence immunoassay system as a comparative method.

### 3.3 Interference Investigation Protocol

Systematic troubleshooting was conducted on March 4, 2026, with all tests performed in duplicate:

Serial Dilution Studies: Patient plasma was diluted 1:4 and 1:8 using manufacturer‐provided assay diluent to assess linearity and distinguish antigen‐excess hook effects from antibody interference [7].

Specific Blocking Tests: Four aliquots were incubated (37°C, 20 min) with:

Polymeric immunoglobulin: 1 mg/mL.

Murine IgG: 100 μg/mL.

Caprine IgG: 100 μg/mL.

Rheumatoid factor blocker (IgM class): 400 μg/mL.

Dose–Response Titration: Murine IgG was titrated at 200,400, and 600 μg/mL to characterize blocking kinetics and assess HAMA titer relative to standard reagent capacity.

Parallel NT‐proBNP Testing: Simultaneous measurement using an alternative platform for discordance analysis.

Note: A parallel true high‐BNP control sample was not available for simultaneous dilution and blocking. Murine IgG stock concentration and dilution effects were accounted for in calculations. Comparative testing using an alternative BNP assay was not available; NT‐proBNP was used as the primary discriminator.

### 3.4 Quality Assurance

All analyses incorporated high, medium, and low concentration quality control materials (CV < 10%). The laboratory maintains ISO15189 accreditation. All procedures were performed by certified clinical laboratory technologists following standardized protocols.

## 4 Results

### 4.1 Reproducibility and Sample Integrity

Repeat testing demonstrated excellent reproducibility (CV = 0.28% between initial and repeat samples), excluding random analytical error. Refrigerated sample retesting showed minimal degradation (4155 pg/mL), consistent with stable interference rather than analytical instability (Table 1).

**TABLE 1:** Serial BNP measurements during hospitalization.

Date	BNP (pg/mL)	Clinical context
2026/2/24	4394	Initial detection
2026/2/26	3464	Repeat testing
2026/2/27	4011	Pre‐echocardiography
2026/3/4	4419 (Test 1)	Systematic investigation
2026/3/4	4407 (Test 2)	Duplicate confirmation

*Note:* BNP was detected by chemiluminescent immunoassay on Pylon PII0099.

### 4.2 Dilution Studies

Serial dilution showed linear response (*R* ^2^ = 0.998) with incomplete recovery. The term “incomplete recovery” indicates that the measured value after dilution was lower than the theoretically expected value, consistent with antibody‐mediated interference rather than genuine analyte. This pattern is consistent with high‐affinity HAMA interference rather than antigen‐excess hook effect (Table 2).

**TABLE 2:** Serial dilution and recovery analysis.

Sample	Measured (pg/mL)	Expected (pg/mL)	Recovery (%)
Neat	4155	—	—
1:4 Dilution	939	1039	90.4
1:8 Dilution	472	519	91
NT‐proBNP	55.8	—	Normal

### 4.3 Specific Interference Identification

Only mouse IgG produced a clear effect (15.7% decrease), while other blockers showed minimal change (< 2%), confirming HAMA as the cause (Table 3). The reduction was modest but highly specific.

**TABLE 3:** Specific interference screening results.

Blocking agent	Concentration	BNP (pg/mL)	Change (%)	Interpretation
None (Control)	—	4155	—	Baseline
Polymer	1 mg/mL	4098	−1.4	Excluded
Murine IgG	100 μg/mL	3501	−15.7	Positive
Caprine IgG	100 μg/mL	4124	−0.8	Excluded
RF Blocker	400 μg/mL	4107	−1.2	Excluded

*Note:* Change rate = (Experimental—valueControl value)/Control value × 100%.

### 4.4 Dose–Response Blocking Analysis

Mouse IgG reduced BNP in a concentration‐dependent but incomplete manner, even at 600 μg/mL (46.1% reduction; residual BNP 2240 pg/mL), indicating very high‐titer HAMA (Table 4).

**TABLE 4:** Dose–response blocking with murine IgG.

Murine IgG (μg/mL)	BNP (pg/mL)	Reduction (%)	Residual interference
0 (Baseline)	4155	—	Severe
200	3235	22.1	Severe
400	2487	40.1	Moderate
600	2240	46.1	Moderate

## 5 Discussion

### 5.1 Mechanism of HAMA Interference

HAMA bridges the murine capture and detection antibodies in the sandwich assay, generating a false signal. The linear dilution pattern is consistent with high‐affinity HAMA interference. The hook effect typically shows non‐linear dilution with paradoxical low values at high concentrations, which was not observed here [7, 12].

Similar cases of HAMA‐induced false‐positive BNP have been reported previously. The present case is distinctive due to the extremely high HAMA titer that resisted standard blocking reagents and required up to 600 μg/mL murine IgG for partial neutralization.

### 5.2 Clinical Implications and Patient Safety

False‐positive BNP results of this magnitude > 4000 pg/mL carry substantial risk for patient harm. Such results may trigger unnecessary emergency cardiac catheterization, inappropriate initiation of intravenous diuretics and vasodilators, prolonged intensive care unit admission, and significant iatrogenic morbidity [10, 11]. In this case, the discordance between severely elevated BNP and completely normal clinical status, corroborated by normal NT‐proBNP and intact cardiac function, prompted appropriate analytical investigation rather than empirical heart failure treatment.

The normal NT‐proBNP 55.8 pg/mL served as a critical discriminator. BNP and NT‐proBNP are co‐secreted from cardiac myocytes in response to wall stress; in genuine heart failure, both markers are concordantly elevated [9, 13]. The profound discordance observed here—BNP > 4000 pg/mL versus NT‐proBNP < 100 pg/mL—is highly suggestive of analytical interference affecting one assay but not the other. This pattern arises because NT‐proBNP assays typically employ different antibody species or detection principles, rendering them less susceptible to the specific HAMA interfering with the BNP assay [8, 9].

### 5.3 Limitations of Current Blocking Strategies

Our systematic blocking studies reveal important limitations in current HAMA mitigation approaches. Commercial heterophilic blocking reagents typically contain 100–200 μg/mL murine IgG or proprietary active blocking formulations [7, 14, 15]. In this case, even 600 μg/mL murine IgG achieved only 46% interference reduction, leaving residual apparent BNP of 2240 pg/mL—still > 20‐fold above the upper reference limit and potentially clinically misleading.

This observation aligns with emerging evidence that high‐titer HAMA can overwhelm standard blocking reagents, particularly in patients with prior exposure to murine monoclonal antibodies for therapeutic or diagnostic purposes [5, 16]. While this patient denied such exposure, HAMA may also develop from environmental contact with rodents, occupational exposure, or certain vaccinations [17, 18]. The incomplete blocking despite supraphysiological murine IgG concentrations suggests this patient's HAMA may possess:

Exceptionally high affinity for murine Fc regions (Kd < 10^−9^ M).

Anti‐idiotypic specificity is not effectively blocked by non‐immune IgG.

IgM class composition with high‐avidity multivalent binding [7, 15].

Recent advances in active blocking reagents—utilizing specific binders that inactivate heterophilic antibodies rather than merely saturating binding sites—may offer superior performance for such high‐titer samples [15]. However, these specialized reagents are not universally incorporated into commercial assays.

### 5.4 Diagnostic Algorithm for Suspected Interference

We propose a 6‐step approach:

1. Clinical–biochemical correlation
2. Preanalytical exclusion
3. Dilution studies: linear but incomplete recovery patterns suggest heterophilic antibody interference; non‐linear patterns suggest hook effects.
4. Platform comparison
5. Specific blocking: species‐specific IgG reduction consistent with interference
6. Quantitative HAMA testing (if available)

Note: Quantitative HAMA ELISA was not performed in this case due to reagent unavailability; however, the marked incomplete blocking even at 600 μg/mL murine IgG indicates high‐titer HAMA.

### 5.5 Clinical Management and Outcome

Recognizing the analytical interference, the clinical team appropriately withheld heart failure‐directed therapy. The patient received standardized diabetes management including insulin pump therapy, antimicrobial therapy for diabetic foot infection, and wound care. Throughout 10 day hospitalization, the patient remained hemodynamically stable without cardiovascular events. Follow‐up at 1 month demonstrated complete wound healing, well‐controlled glycemia, and absence of heart failure symptoms—further confirming the spurious nature of the initial BNP elevation.

### 5.6 Limitations

1. A true high‐concentration BNP control sample was not available for parallel dilution and blocking experiments; thus, matrix effects cannot be fully excluded.
2. Quantitative HAMA ELISA was not performed due to reagent unavailability, although high‐titer HAMA was strongly implied by incomplete blocking even at 600 μg/mL murine IgG.
3. An alternative BNP assay using a different platform was not used for direct comparison; NT‐proBNP was used as the main discriminator.
4. The 15.7% reduction by murine IgG was modest and did not meet the 50% threshold sometimes cited for interference confirmation. However, the high specificity (no effect with polymer, goat IgG, or RF blocker) supports HAMA as the causal agent.

## 6 Conclusions

This case report documents extreme HAMA interference causing false‐positive BNP exceeding 4000 pg/mL in a patient without cardiac dysfunction. The systematic investigation—incorporating serial dilutions, specific blocking studies, and dose–response titration—demonstrates that standard heterophilic blocking reagent concentrations may be insufficient for high‐titer samples. Key clinical insights include:

1. Discordance Recognition: Profound elevation of BNP with normal NT‐proBNP in a clinically stable patient should immediately prompt consideration of analytical interference
2. Systematic Investigation: Linear dilution patterns with incomplete recovery, combined with specific blocking by murine IgG, confirm HAMA interference.
3. Reagent Limitations: Conventional blocking reagents may fail to neutralize high‐titer HAMA, necessitating alternative verification strategies.
4. Patient Safety: Awareness of this phenomenon prevents unnecessary invasive procedures, inappropriate medication, and psychological distress.

Clinical laboratories and practitioners must maintain vigilance for heterophilic antibody interference, particularly in sandwich immunoassays employing murine monoclonal antibodies. When biochemical results discord with clinical presentation, systematic analytical investigation—not empirical treatment—should guide patient management.

## Author Contributions

Huicong Yang: conceptualization, methodology, formal analysis, investigation, writing – original draft. Yuanhai Zheng: data curation, clinical sample collection, investigation, validation, writing – review and editing. Zhenhua Zeng: resources, laboratory testing, data acquisition, validation. Haimin Gao: clinical data curation, patient care, writing – review and editing. Jinzhi Wu: resources, methodology consultation, writing – review and editing. Ayang Wu: supervision, project administration, funding acquisition, writing – review and editing, corresponding author. Jie Lin: supervision, conceptualization, methodology guidance, writing – review and editing, corresponding author. All authors have read and approved the final manuscript.

## Funding

This study was supported by Fujian Provincial Natural Science Foundation of China (2023J011817).

## Ethics Statement

This case report was approved by the Ethics Committee of Zhangzhou Affiliated Hospital of Fujian Medical University (2026GAM001).

## Consent

Written informed consent was obtained from the patient for publication of this case report and any accompanying data.

## Conflicts of Interest

The authors declare no conflicts of interest.

## Data Availability

The data that support the findings of this study are available on request from the corresponding author. The data are not publicly available due to privacy or ethical restrictions.
